# Supplementary figures and images for: The small non-coding RNA Vaultrc5 is dispensable to mouse development
Source: bioRxiv. 2024 Jun 5:2024.06.01.596958. Preprint. [Version 2] doi: 10.1101/2024.06.01.596958 (PMC11185573; doi:10.1101/2024.06.01.596958)

A

chr18:36,953,398-36,962,129

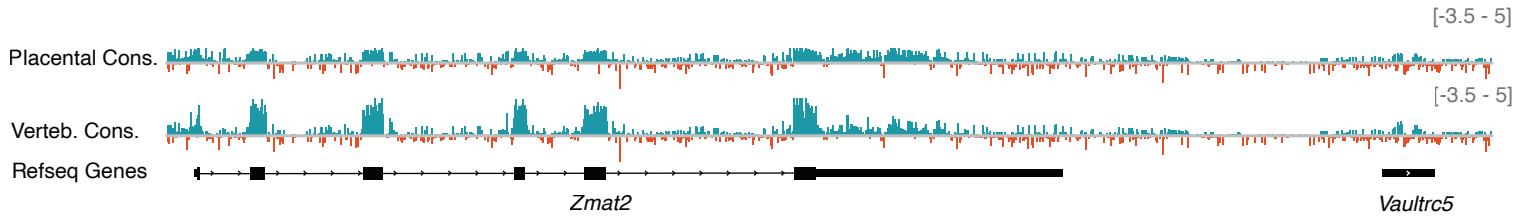

B

chr18:36,961,273-36,961,903

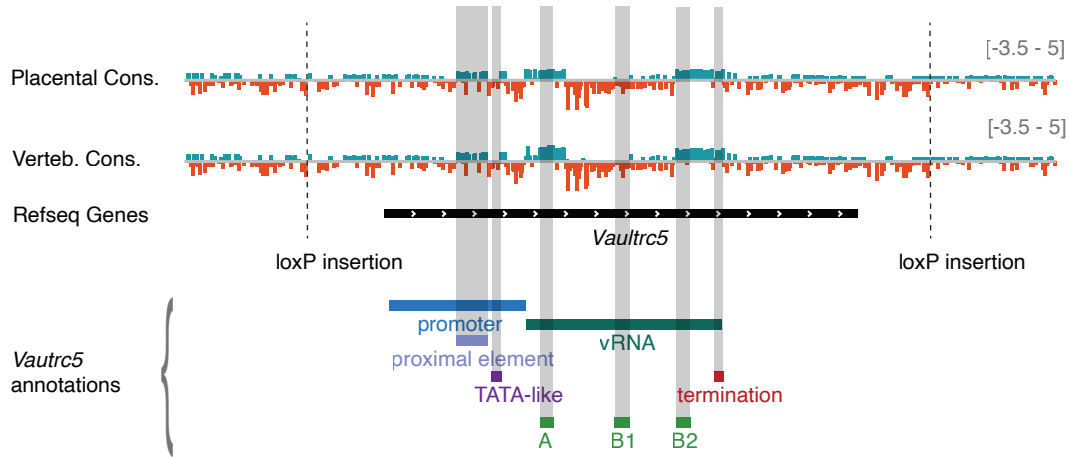

C

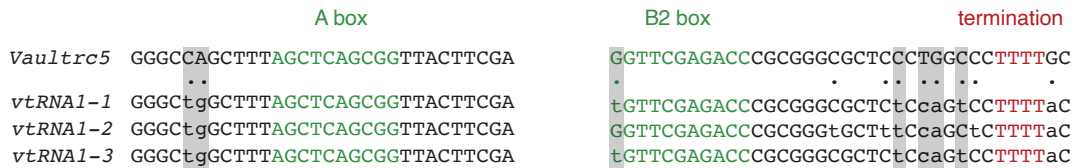

D

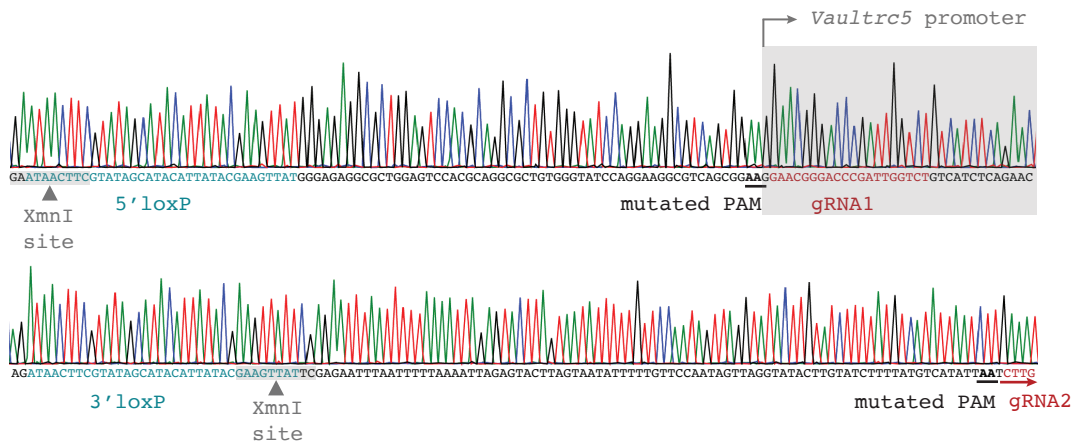

Supplement: Supplement 1 — Supplementary Figure 1. Conservation and targeting of the mouse Vaultrc5 locus. (A) Genome browser view of the syntenic region containing the Vaultrc5 and its conserved upstream neighbor Zmat2. PhyloP basewise conservation scores for Placental mammals and for Vertebrates, as well as UCSC Refseq gene annotations are shown. (B) As in (A) but zoomed in on Vaultrc5 locus. Vertical doted lines highlight the position of the inserted loxP sites in the conditional allele. Position of known regulatory elements are shown below. (C) sequence alignment of the conserved regions between Vaultrc5 and the three human vtRNA1 genes which overlap the A and B2 boxes (in green) and the termination sequence (in red). Dots highlight the nucleotides that differ between the murine gene and at least one of the human vtRNA1 genes. Grey boxes highlight the nucleotides that differ between Vaultrc5 and vtRNA1–1. (D) Sanger sequencing of the integrated donor template showing the position of the loxP sites, the XmnI restriction sites, and the PAMs of the gRNAs used. [file media-1.pdf]

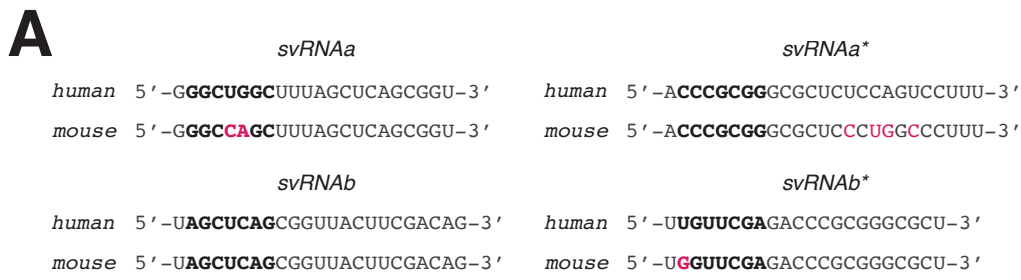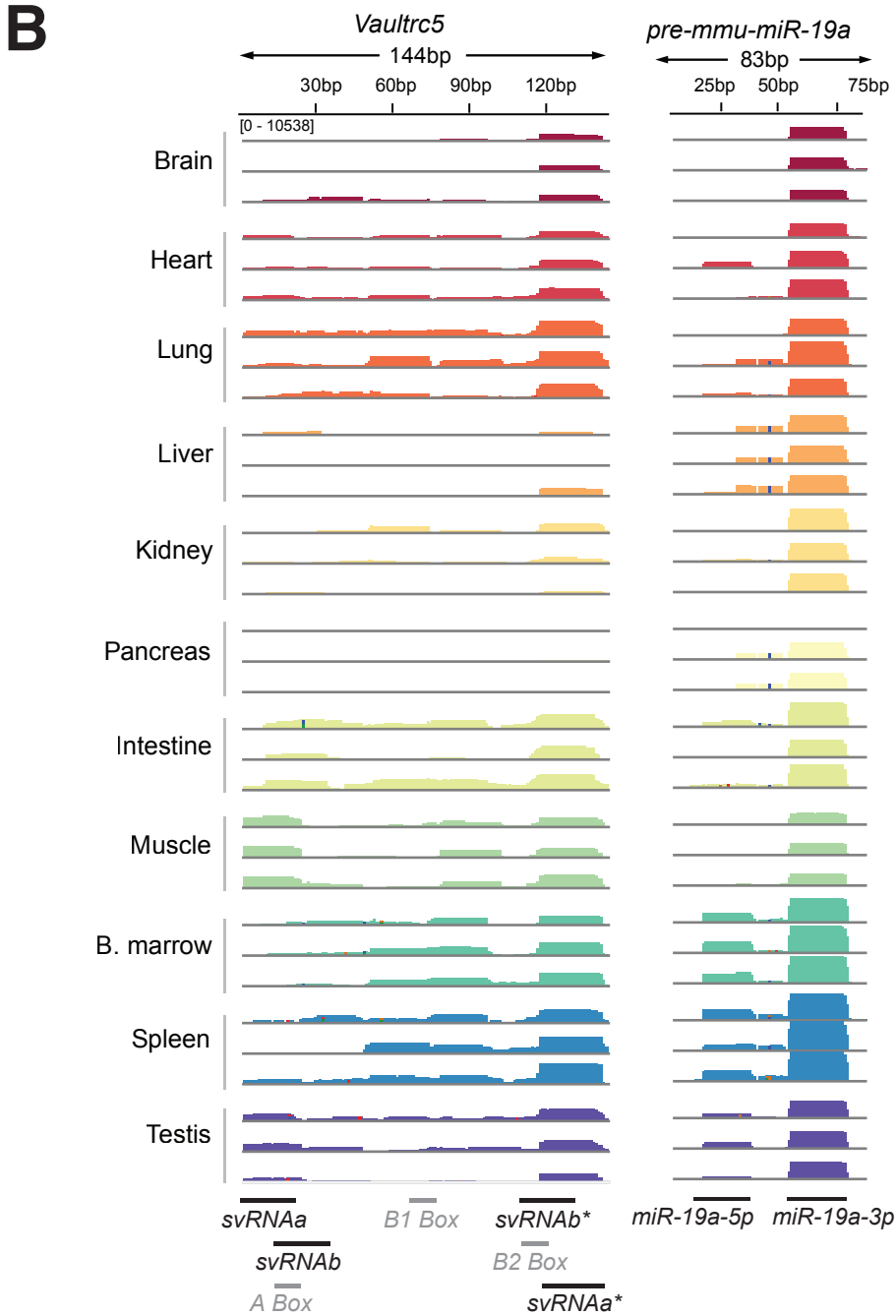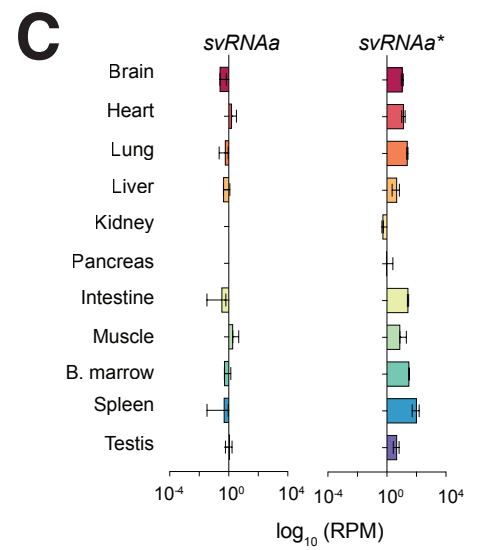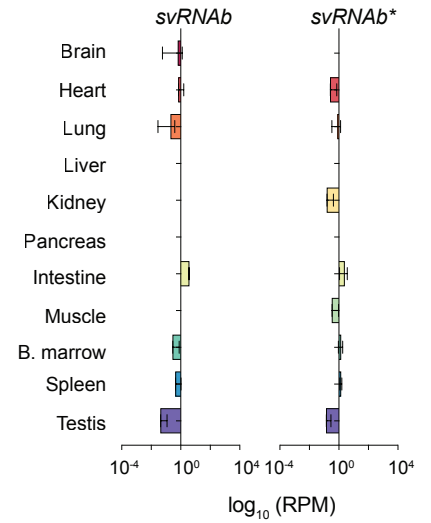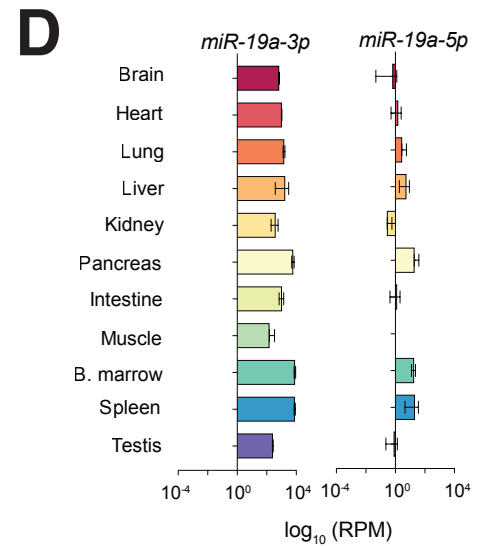

Prajapat Supplementary Figure 2

Supplement: Supplement 2 — Supplementary Figure 2. Analysis of putative miRNAs derived from the Vaultrc5 locus. (A) Comparison of the predicted human and mouse vaultRNA-derived miRNAs. The seed-sequence is highlighted in bold. Nucleotides that differ between the two species are highlighted in pink (B) Genome browser view of sequencing reads over Vaultrc5 and pre-mmu-miR-19a loci in multiple adult mouse tissues in triplicate sequencing experiments (C) Abundance of reads (as RPM) for predicted vaultRNA-derived fragments. Error bars represent standard deviation between triplicate experiments. (D) As in (C) but for miR-19a-3p and miR-19a-5p. [file media-2.pdf]
