# Supplementary material for: The small non-coding RNA Vaultrc5 is dispensable to mouse development
Source: bioRxiv. 2024 Jun 5:2024.06.01.596958. Preprint. [Version 2] doi: 10.1101/2024.06.01.596958 (PMC11185573; doi:10.1101/2024.06.01.596958)
Supplement: Supplement 3 — Supplementary Figure 3. Complete blood counts for Vautrc5−/− and Vautrc5−/− animals. Cell count for White blood cells (A), Red blood cells (B), Platelets, (C). Assessed hemoglobin levels by Hematocrit (D), Mean Red Blood Cell volume (E), Red Blood Cell distribution width (F), Hemoglobin (G), Mean cell hemoglobin (H), Mean cell hemoglobin concentration (I). Error bars represent standard deviation between biological replicates. p-values were calculated with a t-test and are highlighted in green when below the significance threshold of 0.05. [file media-3.pdf]

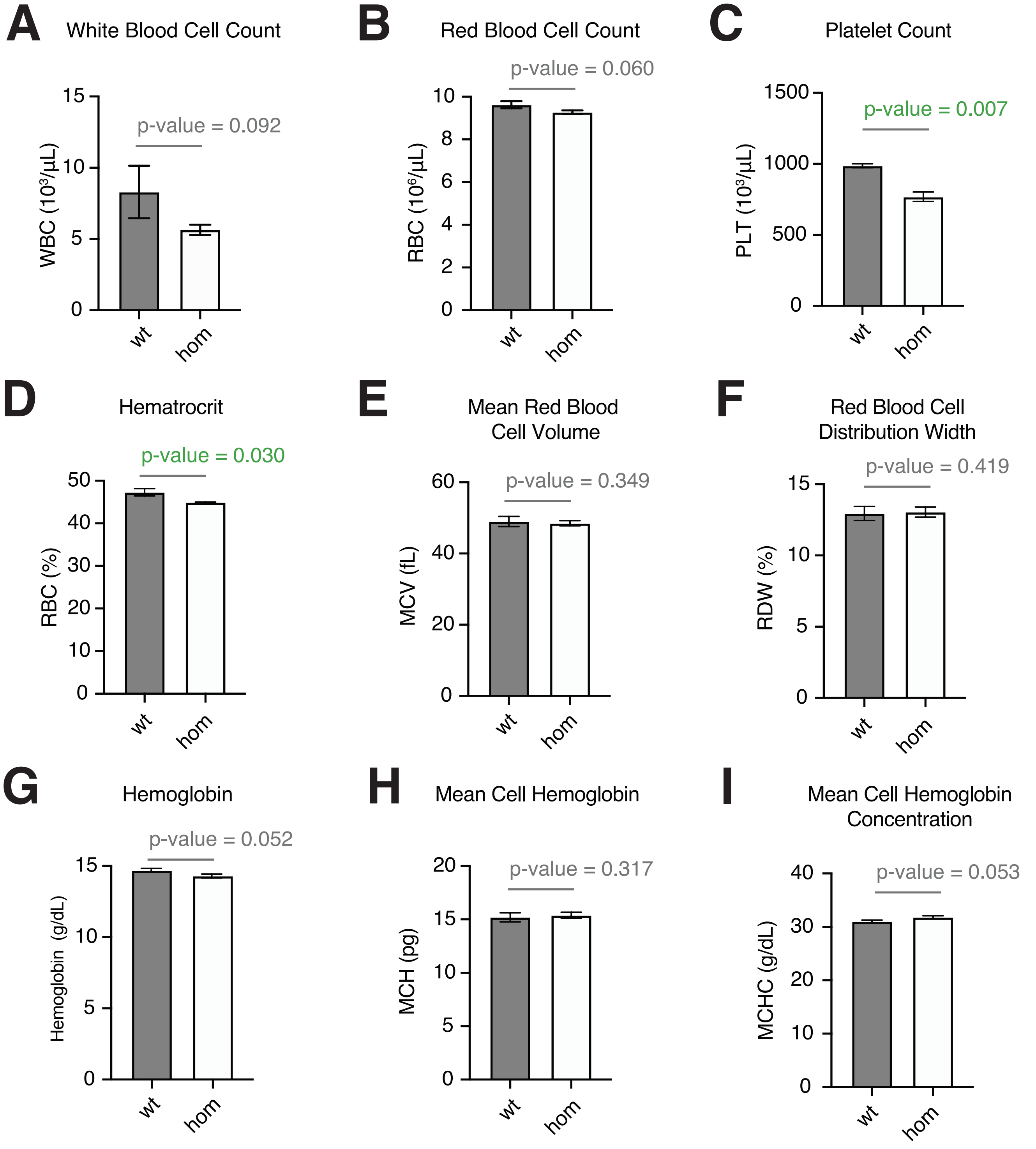

Prajapat Supplementary Figure 3
